# Homoharringtonine regulates the alternative splicing of Bcl-x and caspase 9 through a protein phosphatase 1-dependent mechanism

**DOI:** 10.1186/s12906-018-2233-6

**Published:** 2018-05-22

**Authors:** Qi Sun, Shiyue Li, Junjun Li, Qiuxia Fu, Zhongyuan Wang, Bo Li, Shan-Shan Liu, Zijie Su, Jiaxing Song, Desheng Lu

**Affiliations:** 1 Guangdong Key Laboratory for Genome Stability & Disease Prevention, Carson International Cancer Center, Department of Pharmacology, Shenzhen University Health Science Center, Shenzhen, 518060 Guangdong China; 2 Key Laboratory of Systems Bioengineering (Ministry of Education), Tianjin University, Tianjin, 300072 China

**Keywords:** Homoharringtonine, Alternative splicing, Bcl-x, Caspase 9, Protein phosphatase 1, Apoptosis

## Abstract

**Background:**

Homoharringtonine (HHT) is a natural alkaloid with potent antitumor activity, but its precise mechanism of action is still poorly understood.

**Methods:**

We examined the effect of HHT on alternative splicing of Bcl-x and Caspase 9 in various cells using semi-quantitative reverse transcriptase-polymerase chain reaction (RT-PCR). The mechanism of HHT-affected alternative splicing in these cells was investigated by treatment with protein phosphatase inhibitors and overexpression of a protein phosphatase.

**Results:**

Treatment with HHT downregulated the levels of anti-apoptotic Bcl-xL and Caspase 9b mRNA with a concomitant increase in the mRNA levels of pro-apoptotic Bcl-xS and Caspase 9a in a dose- and time-dependent manner. Calyculin A, an inhibitor of protein phosphatase 1 (PP1) and protein phosphatase 2A (PP2A), significantly inhibited the effects of HHT on the alternative splicing of Bcl-x and Caspase 9, in contrast to okadaic acid, a specific inhibitor of PP2A. Overexpression of PP1 resulted in a decrease in the ratio of Bcl-xL/xS and an increase in the ratio of Caspase 9a/9b. Moreover, the effects of HHT on Bcl-x and Caspase 9 splicing were enhanced in response to PP1 overexpression. These results suggest that HHT-induced alternative splicing of Bcl-x and Caspase 9 is dependent on PP1 activation. In addition, overexpression of PP1 could induce apoptosis and sensitize MCF7 cells to apoptosis induced by HHT.

**Conclusion:**

Homoharringtonine regulates the alternative splicing of Bcl-x and Caspase 9 through a PP1-dependent mechanism. Our study reveals a novel mechanism underlying the antitumor activities of HHT.

## Background

Alternative splicing of mRNA is a key molecular event that allows the generation of multiple mRNAs from a single gene, coding for protein isoforms with different structural and functional properties. Approximately 90% of human genes produce more than one transcript through alternative splicing [1]. This process is highly regulated by a complex interplay between *trans*-splicing factors and *cis*-responsive elements within exons and surrounding introns in normal growth and development [2–4]. Dysregulation of alternative splicing has been found to be associated with various human diseases, including cancer [5–8].

Programmed cell death or apoptosis is a common mechanism to eliminate unnecessary or damaged cells in the development and homeostasis of multicellular organisms. The balance between cell proliferation and apoptosis plays an important role in the control of tissue homeostasis. A failure in apoptosis can lead to the development of neoplasia. Abnormal expressions of apoptosis-related factors are frequently associated with resistance to apoptosis. It is widely observed that many apoptotic genes encode for splice variants with opposing effects on apoptotic regulation. Bcl-x is an anti-apoptotic member of the Bcl-2 gene family and plays a critical role in regulating apoptosis in mammalian cells. Alternative splicing of the Bcl-x gene generates two protein isoforms with opposing functions, anti-apoptotic Bcl-xL and pro-apoptotic Bcl-xS. Relative expressions of these two isoforms control the susceptibility of cells to apoptotic stimuli [9–11]. Caspase 9 is one of the most important initiators of the intrinsic apoptotic pathway and is activated upon the formation of the Apaf-1/cytochrome c complex, termed the apoptosome [12]. The pro-apoptotic Caspase 9a and anti-apoptotic Caspase 9b are derived from the alternative splicing of the Caspase 9 gene. The Caspase 9b isoform lacks catalytic activity and acts as an endogenous inhibitor of Caspase 9a by interfering with the formation of a functional apoptosome complex between Apaf-1 and Caspase 9 [13, 14]. The alternative splicing of Bcl-x and Caspase 9 can be regulated by several small molecules. Chalfant et al. demonstrated the ability of ceramide to induce pro-apoptotic Bcl-xS and Caspase 9a through activation of alternative splicing [15]. Emetine, an inhibitor of protein synthesis, was shown to be able to regulate the alternative splicing of Bcl-x and Caspase 9 in tumor cells [16, 17]. Chang et al. showed that the antihypertensive drug amiloride could modulate the alternative splicing of various cancer genes, including Bcl-x, HIPK3, and BCR/ABL, in leukemia cells [18, 19]. The manipulation of the alternative splicing of Bcl-x and Caspase 9 may have therapeutic potential in cancer treatment.

Homoharringtonine (HHT) is a natural alkaloid derived from various Cephalotaxus species and has been used in the treatment of hematological malignancies for the past 30 years in China [20]. In 2012, the US FDA approved the use of HHT for treating patients with chronic or accelerated phase chronic myeloid leukemia (CML) [21]. In multiple myeloma (MM) cells, HHT showed anti-myeloma effect with concomitant targeting of the myeloma-promoting molecules, Mcl-1, XIAP, and beta-catenin [22]. Recently, Chen et al. reported that HHT could inhibit the proliferation of acute myeloid leukemia (AML) cells by targeting Smad3/TGF-β pathway [23]. Although HHT has been shown to exert its anticancer activity partly through inhibition of protein synthesis and promotion of apoptosis, its detailed molecular mechanism remains unknown. In this study, we demonstrated for the first time that HHT could regulate the alternative splicing of Bcl-x and Caspase 9 pre-mRNA in several human cancer cells. We further demonstrated that the effect of HHT on alternative splicing is mediated by PP1.

## Methods

### Compounds

**Fig. 1:**
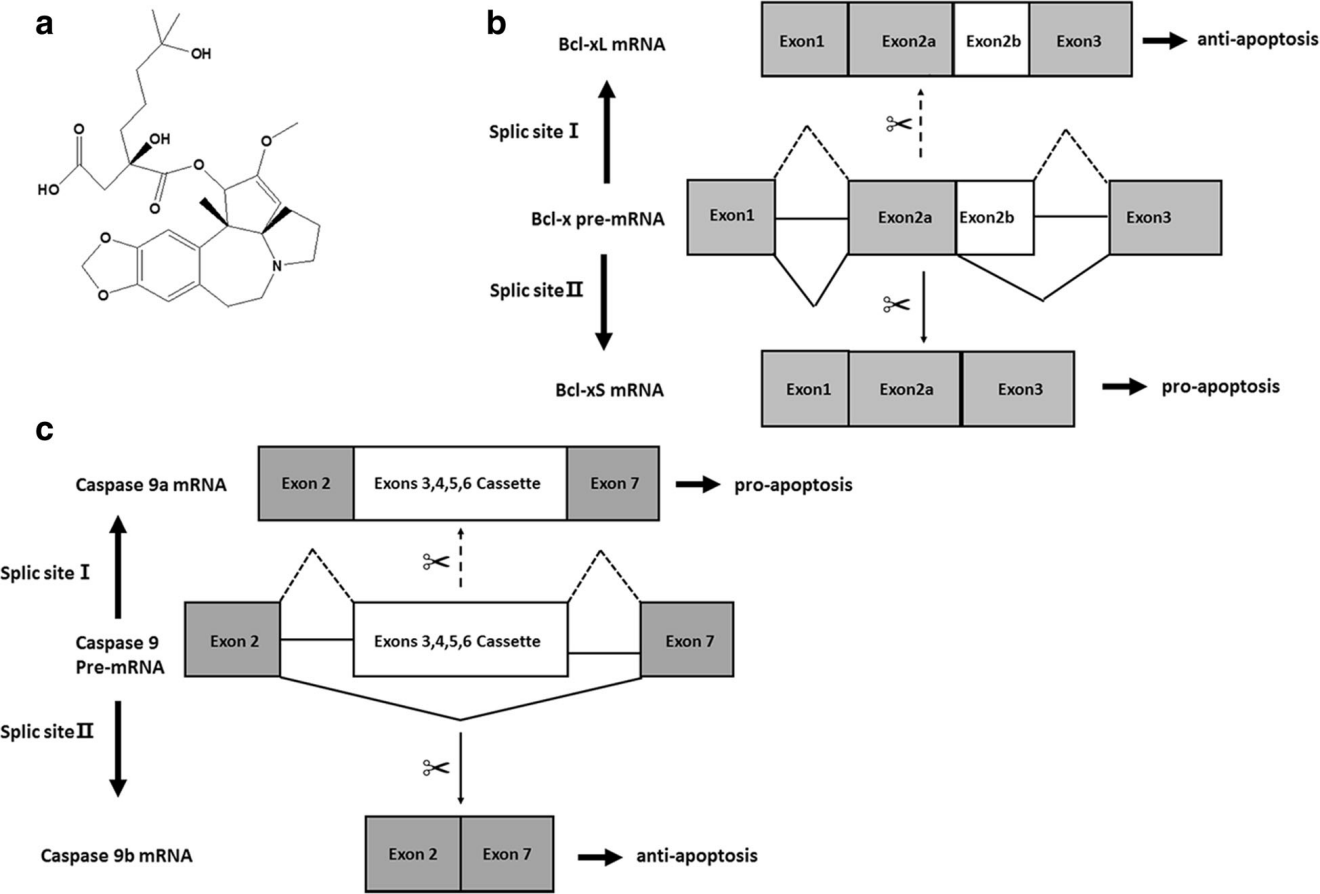
Alternative splicing of Bcl-x and Caspase 9. **a** Chemical structure of HHT. **b** Schematic splicing of Bcl-x pre-mRNA generating Bcl-xL and Bcl-xS mRNA via alternative 5′ splice site selection. **c** Schematic splicing of Caspase 9 pre-mRNA generating Caspase 9a and Caspase 9b mRNA via cassette exon (3, 4, 5, 6) inclusion and exclusion, respectively

Homoharringtonine (Fig. 1a) was purchased from Sigma (St. Louis, MO, USA). Phosphatase inhibitors calyculin A and okadaic acid were purchased from Cell Signaling Technology (Boston, MA, USA).

### Cell culture

Human cancer cell lines MCF7, A549, UACC903 were purchased from the Chinese Academy of Sciences Cell Bank (Shanghai, China). Human breast cancer MCF7, MCF-7/Bobi and MCF-7/PP1 cells were maintained in Dulbecco’s modified Eagle’s media (DMEM), supplemented with 10% fetal bovine serum (FBS), L-Glutamine and penicillin-streptomycin. Adenocarcinoma lung cancer cells A549 were grown in DMEM/nutrient mixture F-12 (Ham) supplemented with 10% FBS, L-Glutamine and penicillin-streptomycin. Human melanoma UACC903 cells were cultured in Roswell Park Memorial Institute (RPMI1640) with 10% FBS, L-glutamine and penicillin-streptomycin. All cultures were maintained in a humidified 5% CO_2_ incubator at 37 °C and routinely passed when 80–90% confluent.

### HHT treatment

Homoharringtonine was dissolved in DMSO, with the stock solution concentration at 5 mM. The stock solution was diluted with the cell-specific media and the final DMSO concentration is < 0.1%. HHT was used at concentrations less than 50% inhibitory concentration (IC_50_) in the studies. At 24 h prior to HHT treatment, the cells were plated in 2 ml of medium in 6-well plates at a density of 200,000 cells/well. The cells were treated with different concentrations of HHT for 24 h for the dose-dependent study. For the time course experiment, the cells were treated with 0.5 μM of HHT for various durations in MCF7 cells.

### Treatment with protein phosphatase inhibitors

MCF7, A549 and UACC903 cells were pretreated with calyculin A (2 nM) or okadaic acid (5 nM) for 1 h, after which the media was removed. Fresh media with HHT was then added to the cells for 24 h. Semi-quantitative RT-PCR was performed to evaluate Bcl-x and Caspase 9 splicing.

### Semi-quantitative RT-PCR

Total RNA was extracted from cultured MCF7, A549 and UACC903 cells using Trizol reagent (Takara, Shiga, Japan) according to the manufacturer’s instruction. Reverse transcription was carried out with 0.5 μg total RNA using the PrimerScript™ RT reagent kit (Takara, Shiga, Japan). After incubation for 1 h at 42 °C, the reactions were terminated by heating at 70 °C for 15 min. To analyze alternative splicing of exon 2 in the Bcl-x gene, 5′ primer to Bcl-x (5′-GAGGCAGGCGACGAGTTTGAA-3′) and 3′ primer (5′-TGGGAGGGTAGAGTGGATGGT-3′) were used for PCR amplification (30 cycles, 94 °C, 30s; 56 °C, 30s; 72 °C, 1 min) with 2xEasy Taq superMix (Transgen Biotech, China). The length of splicing variants of Bcl-xL and Bcl-xS are 460 bp and 271 bp respectively. To analyze alternative splicing of exon 3, 4, 5, 6 in the Caspase 9 gene, 5′ primer to Caspase 9 (5′- GCTCTTCCTTTGTTCATCTCC -3′) and 3′ primer (5′- CATCTGGCTCGGGGTTACTGC -3′) were used for PCR amplification (30 cycles, 94 °C, 30s; 54 °C, 30s; 72 °C, 1 min). The length of splicing variants of Caspase 9a and Caspase 9b are 742 bp and 292 bp, respectively. PCR products were separated and analyzed on agarose gels, with the bands of the Bcl-x and Caspase 9 splicing variants being confirmed by DNA sequencing.

### Western blotting

MCF7, A549, UACC903 cells were treated with control or HHT for 24 h, then harvested and sonicated in lysis buffer buffer (20 mM Tris·HCl pH 7.4, 150 mM NaCl, 1 mM EDTA, 1 mM EGTA, 1% Triton X-100, 2.5 mM sodium pyrophosphate, 1 mM β-glycerol phosphate, 1 mM sodium orthovanadate, 2 μg/mL leupeptin, and 1 mM PMSF). Equal amount of proteins were resolved by SDS/PAGE, followed by immunoblotting with a specific Bcl-xL antibody (Cell Signaling Technology).

### PP1 cloning, lentiviral vector production and infection

In order to construct the stable overexpression of PP1 in MCF7 cells, a lentiviral vector pBobi was used for gene delivery. The human PP1 gene (XM_001348279.1) was amplified by PCR from a complementary deoxyribonucleic acid (cDNA) library. Following the addition of 10 μl of the PCR products onto a 1% agarose gel with ethidium bromide (0.5 mg/ml) and electrophoresis, images were captured by ultraviolet transillumination. The plasmid was doubly digested with Kpnl and Xba1 (Thermo Fisher Scientific, San Jose, CA, USA). The PCR products and plasmid were purified and ligated, and the resultant mixture was transformed into competent *Escherichia coli* DH5α cells (Transgen Biotech, China). Clones were selected for PCR validation and the recombinant plasmid was extracted for sequencing. The lentivirus packaging system is consisted of 3 plasmids: pMDLg/pRRE, pRSV-Rev, and pVSV-G. To produce the PP1 lentivirus, the recombinant pBobi vector was cotransfected with pMDLg/pRRE, pRSV-Rev, pVSV-G into HEK293T cells. The culture supernatants containing the virus were collected 48 h and 72 h after transfection. For infection with lentivirus, MCF7 cells were cultured with the lentiviral solution for 24 h in the presence of 1 μg/mL Polybrene (Sigma, St. Louis, MO, USA). The resulting cell line was named MCF7-PP1. The control cell line MCF7-Bobi was transfected with an empty vector.

### Annexin V-PE /7-Aminoactinomycin D (7-AAD) staining

MCF7-Bobi and MCF7-PP1 cells treated with 5 μM HHT for 24 h were collected and incubated with Annexin V-PE and 7-Aminoactinomycin D (7-AAD) fluorescein solutions (Multi Sciences, China) according to the manufacturer’s protocol. The FACSCalibur™ (BD Biosciences, San Jose, CA, USA) fluorescent-activated cell-sorting (FACS) instrument was used for quantitative fluorescent sorting, and FlowJo v10.0.8 (TreeStar Inc., Ashland, OR, USA) was used for subsequent analysis.

### Statistical analyses

Student’s t-test was used to compare means between groups and all data are represented as the mean ± SEM. Differences at *P* < 0.05 were considered statistically significant.

## Results

### Homoharringtonine regulates the alternative splicing of Bcl-x and caspase 9 pre-mRNA in breast cancer MCF7 cells

**Fig. 2:**
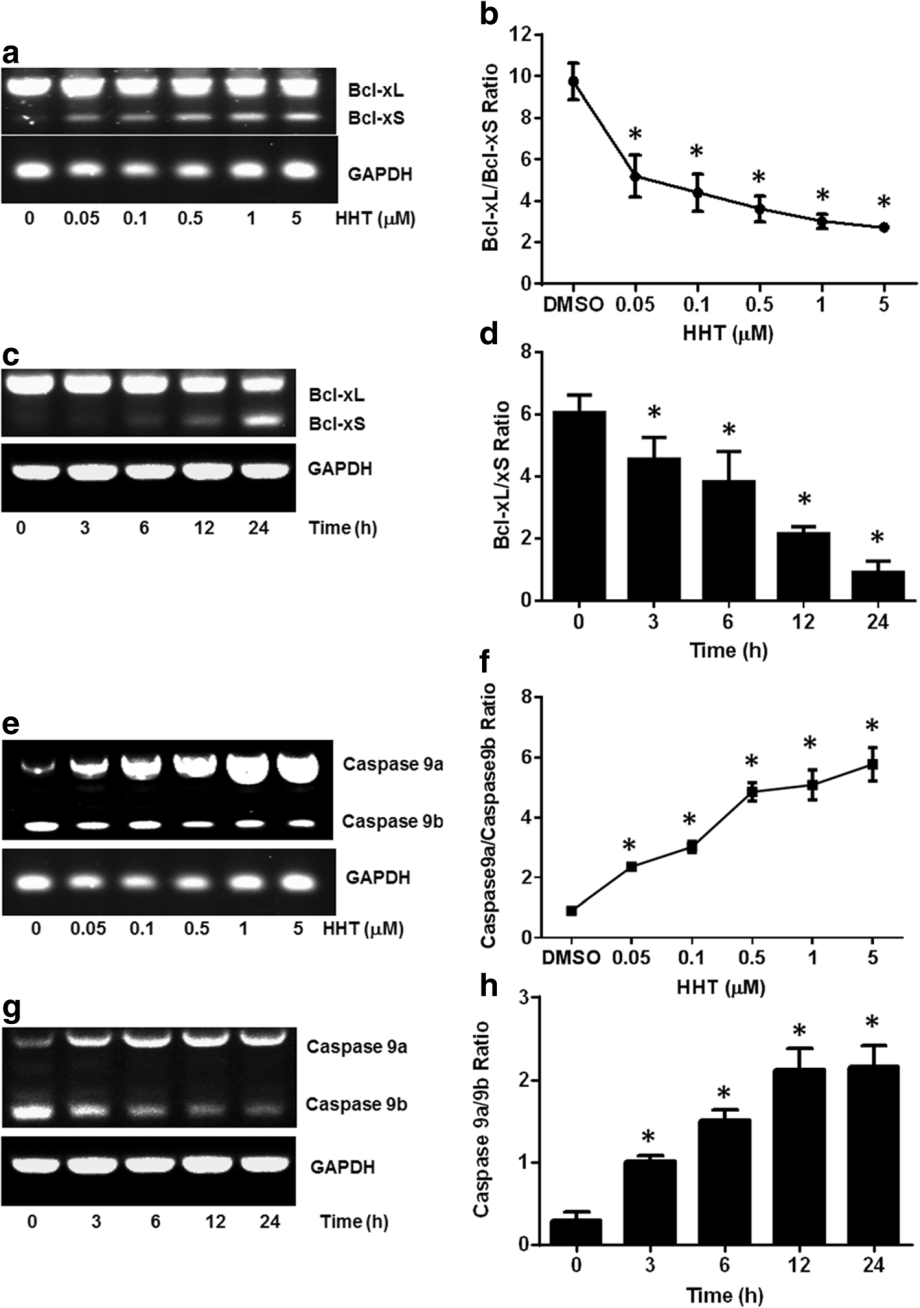
HHT regulates Bcl-x and Caspase 9 splicing in MCF7 cells. MCF7 cells were treated with HHT. Total RNA was extracted and analyzed by semi-quantitative RT-PCR for the alternative splicing of Bcl-x and Caspase 9. **a** Decrease of Bcl-xL and increase of Bcl-xS is correlated with HHT concentration. **b** Densitometric analysis of the ratio of Bcl-xL/xS (*P < 0.05 compared to cells treated with control). **c** and **d** Cells were treated with 0.5 μM HHT for different durations. Semi-quantitative RT-PCR was performed to quantify Bcl-x splicing (*P < 0.05 compared to cells at 0 h). **e** Decrease of Caspase 9b and increase of Caspase 9a is correlated with HHT concentration. **f** Densitometric analysis of the ratio of Caspase 9a/9b (*P < 0.05 compared to cells treated with control). **g** and **h** Cells were treated with 0.5 μM HHT for different durations. Semi-quantitative RT-PCR was then performed to quantify the alternative spliced products of Caspase 9 (*P < 0.05 compared to cells at 0 h)

We explored the effects of some natural products on the alternative splicing of Bcl-x using semi-quantitative RT-PCR in MCF7 cells. Alternative splicing of the Bcl-x gene generates Bcl-xL and Bcl-xS variants by using the alternative 5′ splice site within exon 2 (Fig. 1b). Our preliminary results indicate that HHT might affect the alternative splicing of Bcl-x. To further validate the effect of HHT on Bcl-x splicing, MCF7 cells were treated with various concentrations of HHT for 24 h (Fig. 2a-b) or with 0.5 μM HHT for different time durations (Fig. 2c-d). We observed a decrease in the ratio of Bcl-xL/xS from 9.9 in the DMSO control to 4.4, 3.4, 3.0, 2.6, and 2.5 with HHT at 0.05, 0.1, 0.5, 1.0, 5.0 μM, respectively (Fig. 2a-b). Moreover, HHT significantly reduced the ratio of Bcl-xL/xS in a time-dependent manner (Fig. 2c-d).

We further tested the effect of HHT on Caspase 9 splicing in MCF7 cells (Fig. 2e-h). The two splice variants of caspase-9 (Caspase 9a and Caspase 9b) can be generated by either the inclusion or exclusion of the exon 3,4,5,6 cassette in the mature caspase-9 mRNA (Fig. 1b). As shown in Fig. 2e and f, HHT treatment increased pro-apoptotic Caspase 9a with a concomitant decrease of the anti-apoptotic smaller Caspase 9b, resulting in an increase in the ratio of the Caspase 9a/9b isoform. Importantly, the effect of HHT on the alternative splicing of Caspase 9 is concentration- (Fig. 2e-f) and time-dependent (Fig. 2g-h).

### The effects of HHT on the alternative splicing of Bcl-x and caspase 9 in A549 and UACC903 cells

**Fig. 3:**
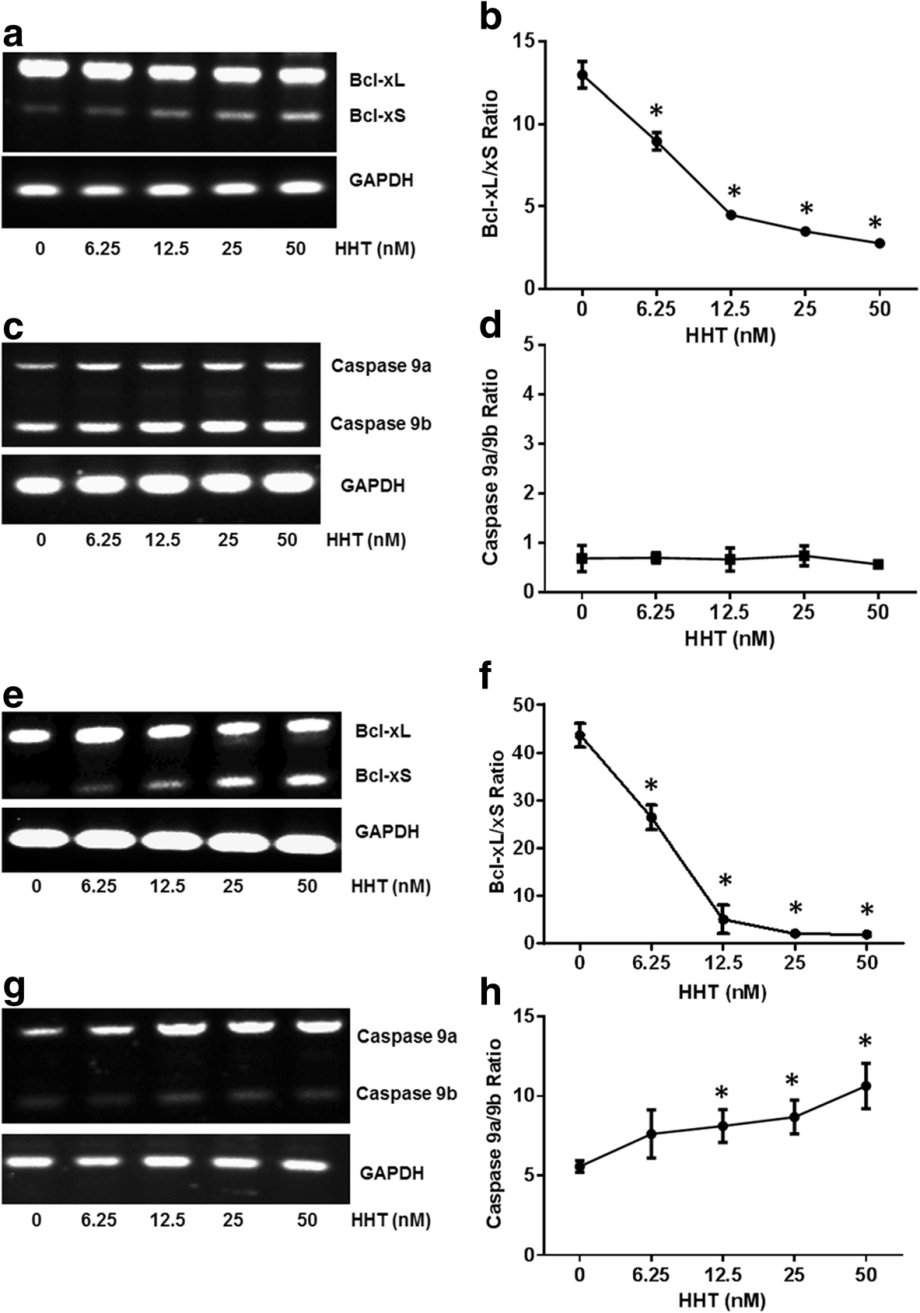
HHT regulates Bcl-x and Caspase 9 splicing in A549 and UACC903 cells. Total RNA was extracted from A549 cells and UACC903 cells treated with HHT. The alternative splicing of Bcl-x and Caspase 9 was then analyzed by semi-quantitative RT-PCR. **a** Semi-quantitative RT-PCR analysis of Bcl-x splicing from A549 cells treated with different concentrations of HHT. **b** Densitometric analysis of the ratio of Bcl-xL/xS in A549 cells treated by HHT (*P < 0.05 compared to cells treated with control). **c** Semi-quantitative RT-PCR analysis of Caspase 9 splicing from A549 cells treated with different concentrations of HHT. **d** Densitometric analysis of the ratio of Caspase 9a/9b in A549 cells treated by HHT. **e** Semi-quantitative RT-PCR analysis of Bcl-x splicing from UACC903 cells treated with different concentrations of HHT. (f) Densitometric analysis of the ratio of Bcl-xL/xS in UACC903 cells treated by HHT (*P < 0.05 compared to cells treated with control). **g** Semi-quantitative RT-PCR analysis of Caspase 9 splicing from UACC903 cells treated with different concentrations of HHT. **h** Densitometric analysis of the ratio of Caspase 9a/9b in UACC903 cells treated by HHT (*P < 0.05 compared to cells treated with control)

To explore whether HHT-induced alternative splicing has potential relevance in cancer treatment, we examined the effect of HHT on the alternative splicing of Bcl-x and Caspase 9 in human non-small cell lung cancer A549 cells and human malignant melanoma UACC903 cells (Fig. 3). A549 and UACC903 cells were significantly more sensitive to HHT. The dramatic change in the alternative splicing of Bcl-x can be achieved with doses much lower than those required to affect Bcl-x splicing in MCF7 cells. As shown in Fig. 3, 50 nM HHT induced profound effects on the alternative splicing of Bcl-x, decreasing the ratio of Bcl-xL/xS from 13.3 to 2.9 in A549 cells (Fig. 3a-b) and from 44.3 to 2.6 in UACC903 cells (Fig. 3e-f). For Caspase 9, 50 nM HHT induced a significant increase in the ratio of Caspase 9a/9b from 6.0 to 12.3 in UACC903 cells (Fig. 3g-h). However, treatment with HHT had no effect on Caspase 9 splicing in A549 cells (Fig. 3c-d), suggesting that the regulation of the alternative splicing of Caspase 9 in response to HHT may be cell line-specific.

### Homoharringtonine decreases the protein expression of Bcl-xL in MCF7, A549 and UACC903 cells

**Fig. 4:**
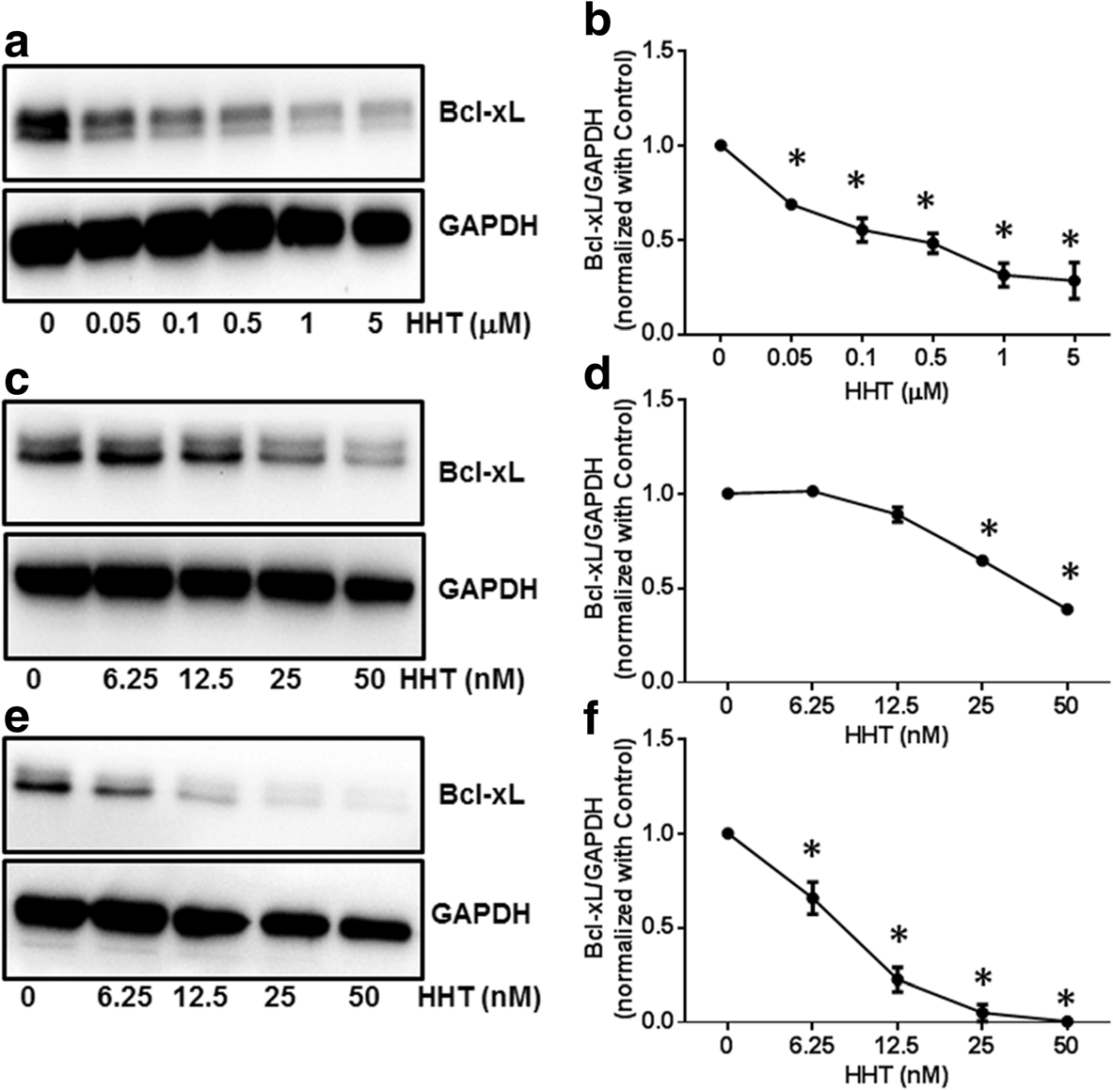
HHT effects on the protein expression of Bcl-xL in MCF7, A549 and UACC903 cells. MCF7 (**a**), A549 (**c**) and UACC903 (**e**) cells were treated with control or HHT for 24 h. Total cell lystates were isolated and assayed for expression by western blotting using a specific Bcl-xL antibody. Densitometric analysis of the Bcl-xL protein expression in MCF7 (**b**), A549 (**d**), UACC903 (**f**) (*P < 0.05 compared to cells treated with control)

To examine the effect of HHT on protein level of Bcl-xL, a Bcl-xL specific antibody was used to detect the protein expression of Bcl-xL in MCF7, A549 and UACC903 cells. Western blotting showed that HHT decreased the protein level of Bcl-xL in a dose-dependent manner (Fig. 4), which is consistent with its effect of downregulating Bcl-xL mRNA level.

### Homoharringtonine exerts its effect on the alternative splicing of Bcl-x and caspase 9 via PP1

**Fig. 5:**
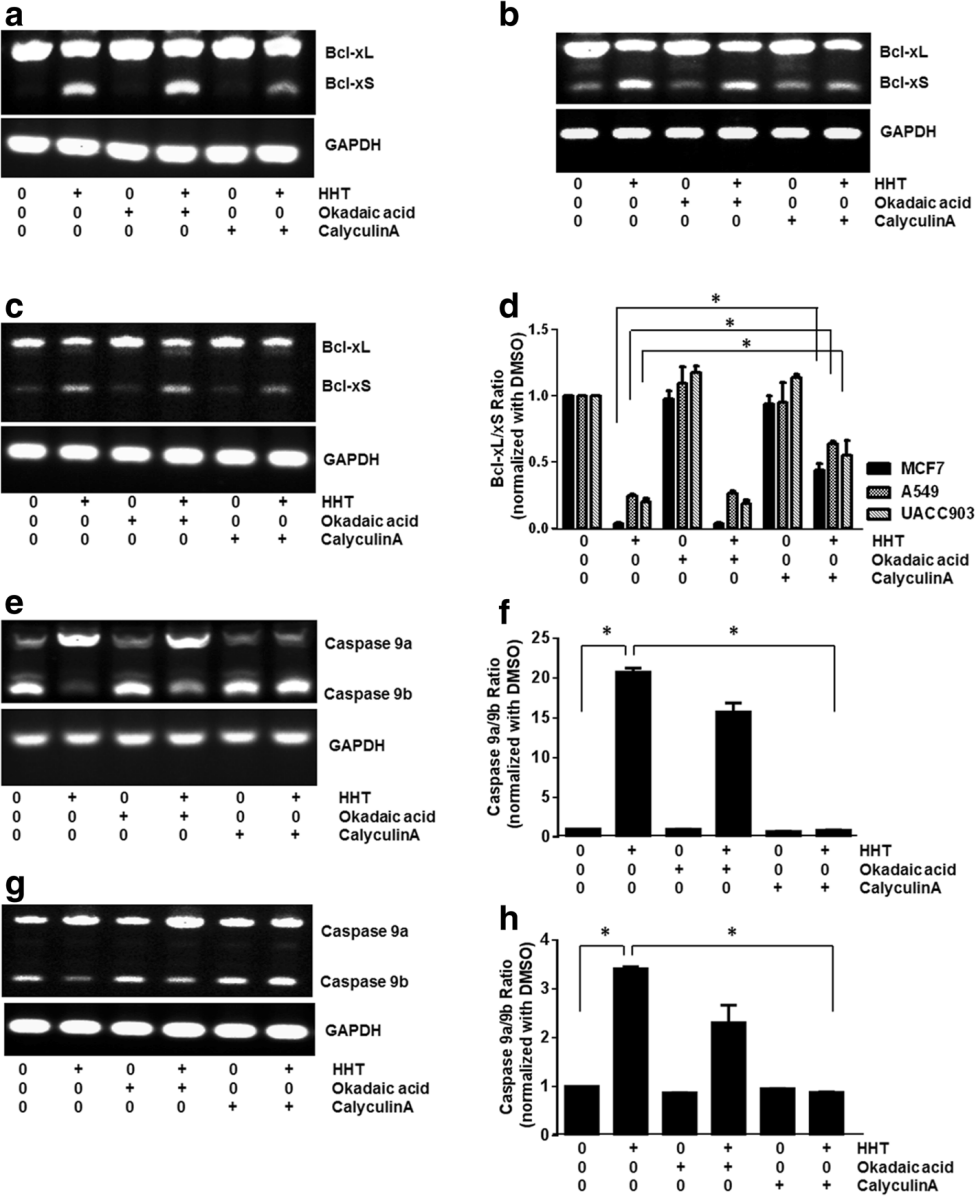
CalyculinA blocks effects of HHT on Bcl-x and Caspase 9 splicing. Cells were pretreated with either 2 nM calyculin A or with 5 nM okadaic acid, and then exposed to the indicated concentration of HHT for 24 h. Semi-quantitative RT-PCR was performed. **a** Effects of calyculin A on HHT-induced Bcl-x splicing in MCF7 cells. **b** Effects of calyculin A on HHT-induced Bcl-x splicing in A549 cells. **c** Effects of calyculin A on HHT-induced Bcl-x splicing in UACC903 cells. **d** Densitometric analysis of the ratio of Bcl-xL/xS in MCF7, A549 and UACC903 cells (*P < 0.05). **e** Effects of calyculin A on HHT-induced Caspase 9 splicing in MCF7 cells. **f** Densitometric analysis of the ratio of Caspase 9a/9b in MCF7 (*P < 0.05). **g** Effects of calyculin A on HHT-induced Caspase 9 splicing in UACC903 cells. **h** Densitometric analysis of the ratio of Caspase 9a/9b in UACC903. +, with HHT, okadaic acid or calyculin A; −, without HHT, okadaic acid or calyculin A (*P < 0.05)

Previous studies showed that ceramide and emetine modulate the alternative splicing of Bcl-x and Caspase 9 by affecting PP1. To test whether PP1 mediates the effects of HHT on the alternative splicing of Bcl-x and Caspase 9, MCF7, A549 and UACC903 cells were pretreated for 1 h with 2 nM calyculin A, an inhibitor of both PP1 and PP2A, or 5 nM okadaic acid, a selective PP2A inhibitor, prior to HHT treatment. Calyculin A partially inhibited the effects of HHT on Bcl-x splicing, while pretreatment with okadaic acid had no effect on Bcl-x splicing in all three cell lines (Fig. 5a-d). These results suggest that PP1 mediates the effects of HHT on the alternative splicing of Bcl-x.

In the case of Caspase 9, calyculin A blocked the effect of HHT on Caspase 9 splicing in MCF7 (Fig. 5e-f) and UACC903 cells (Fig. 5g-h). However, okadaic acid pretreatment slightly relieved the effects of HHT on Caspase 9 splicing in MCF7 and UACC903 cells, suggesting that PP2A may also be involved in the HHT-mediated splicing of Caspase 9.

### Overexpression of PP1 enhances the effects of HHT on Bcl-x and caspase 9 splicing and sensitizes MCF7 cells to apoptosis induced by HHT

**Fig. 6:**
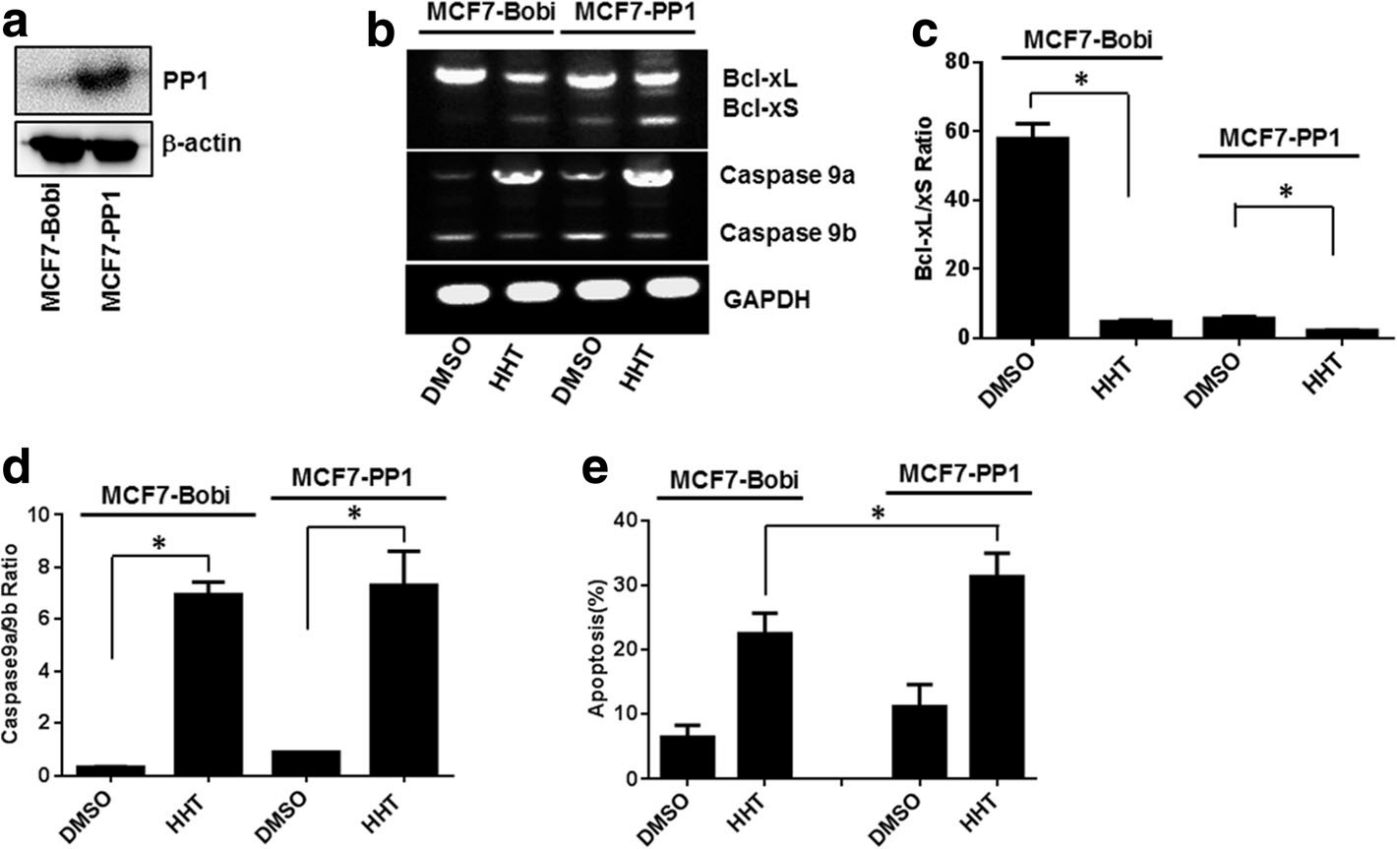
Overexpression of PP1 increases alternative splicing and promotes tumor cell apoptosis induced by HHT. A stable MCF7 cell line overexpressing PP1 was constructed with a lentiviral expression system. MCF7-Bobi and MCF7-PP1 cells were treated with 0.5 μM HHT. Total RNA was extracted and analyzed by semi-quantitative RT-PCR for the splicing of Bcl-x and Caspase 9. **a** Overexpression of PP1 in MCF7 cells. **b** Semi-quantitative RT-PCR analysis of Bcl-x and Caspase 9 splicing from MCF7-Bobi and MCF7-PP1 cells treated with 0.5 μM HHT. **c** and **d** Ratio of Bcl-xL/xS and Caspase 9a/9b in MCF7-Bobi and MCF7-PP1 cells (*P < 0.05). **e** Apoptosis induced by 5 μM HHT in MCF7-Bobi and MCF7-PP1 cells (*P < 0.05)

To verify the role of PP1 in HHT-mediated alternative splicing, we constructed a stable MCF7 cell line overexpressing PP1 using a lentiviral system (Fig. 6a). Significant changes in the alternative splicing of Caspase 9 and Bcl-x were observed in the MCF7 cells overexpressing PP1, with a decrease in the ratio of Bcl-xL/xS and an increase in the ratio of Caspase 9a/9b compared with the MCF7 control cells (Fig. 6b-d). Moreover, the effects of HHT on Bcl-x and Caspase 9 splicing were further enhanced in response to PP1 overexpression.

We next examined the effect of HHT on apoptosis in the PP1 overexpressing MCF7 cell line and parental MCF7 cell line. Cellular apoptosis was evaluated by measurement of the exposure of phosphatidylserine on the cell membrane by using Annexin V-PE and 7-AAD staining. Figure 6e showed that HHT induced apoptosis in MCF7 cells. About 22.54% cells were apoptotic when treated with HHT, while the apoptotic rate was 6.44% in the untreated cells (Fig. 6e). Overexpression of PP1 also induced apoptosis and sensitized MCF7 cells to apoptosis induced by HHT. Treatment with HHT induced an apoptotic rate of 31.41% in the PP1 overexpressing MCF7 cells (Fig. 6e).

## Discussion

**Fig. 7:**
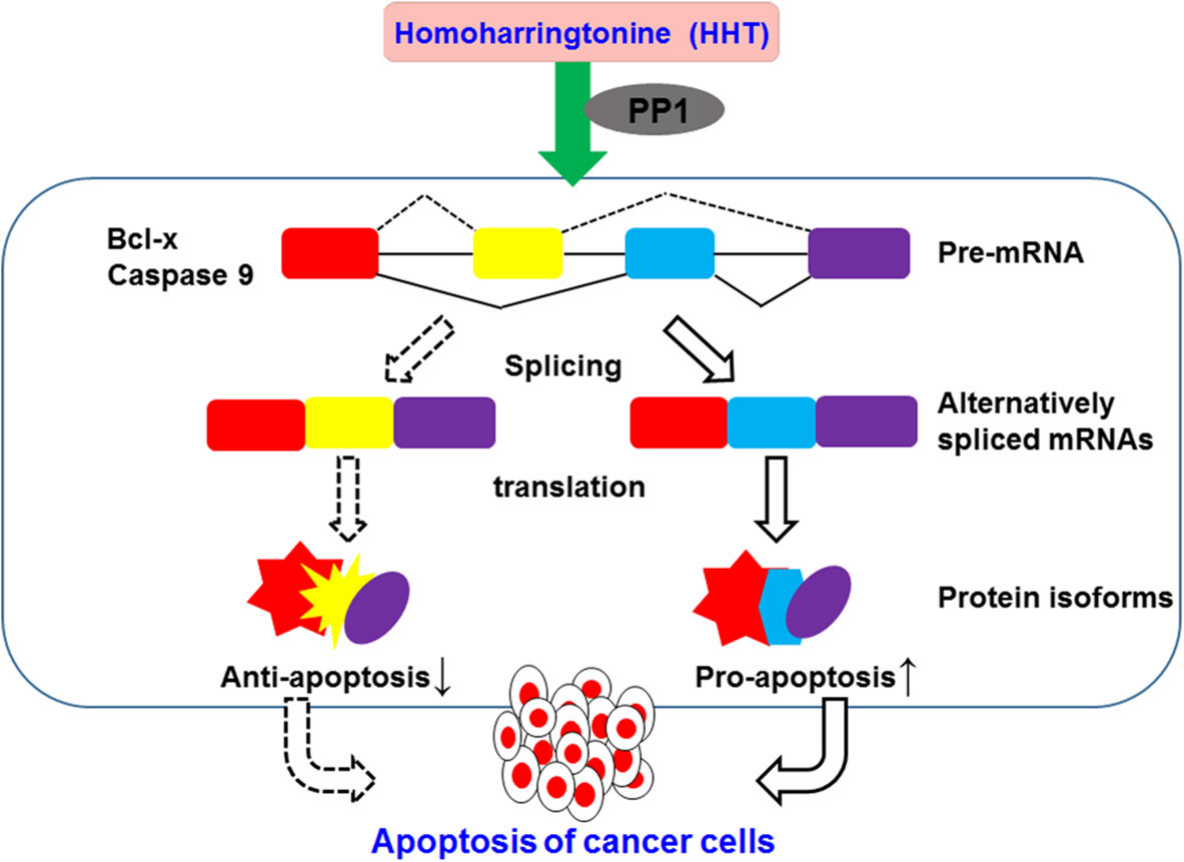
A novel mechanism underlying the antitumor activities of HHT. Homoharringtonine regulates the alternative splicing of Bcl-x and Caspase 9 through a PP1-dependent mechanism, resulting in a decreased expression of anti-apoptotic Bcl-xL and Caspase 9b with a concomitant increase in the levels of pro-apoptotic Bcl-xS and Caspase 9a in various cancer cells

Homoharringtonine has been widely used to treat hematopoietic malignant disorders, such as AML and CML [24, 25]. A number of clinical studies have confirmed its clinical therapeutic effect. However, the mechanism underlying the antitumor activities of HHT is still poorly understood. HHT is a potent apoptosis inducer in a variety of leukemia cells. The apoptosis-inducing ability of HHT might account for its main therapeutic potential in the treatment of patients with leukemia. Increasing evidence showed that HHT could induce cell apoptosis through various intrinsic and extrinsic apoptotic pathways, including down-regulation of myeloid cell leukemia-1 (Mcl-1), XIAP and survivin [22, 26], and the activation of caspase-3, caspase-8, caspase-9 and PARP [27, 28]. Several studies have demonstrated that HHT could induce apoptosis via inhibition of protein synthesis and down-regulation of Mcl-1 in chronic lymphocytic leukemia and myeloid leukemia cells [26, 29, 30]. Meng et al., reported that HHT inhibited AKT phosphorylation and downregulated the expression of several AKT target genes, including NF-κB, XIAP, cIAP and Cyclin D1 in MM cells [31]. Recently, HHT has been shown to induce apoptosis and suppress STAT3 via IL-6/JAK1/STAT3 signal pathway in Gefitinib-resistant lung cancer cells [32]. Moreover, Yin et al. noted that HHT treatment significantly decreased the levels of Bcl-xL and identified Bcl-xL as a dominant anti-apoptotic protein that inhibits HHT-induced apoptosis in leukemia cells [33]. In the present study, we showed that HHT could regulate the alternative splicing of Bcl-x and Caspase 9, resulting in a decrease in the levels of anti-apoptotic Bcl-xL and Caspase 9b mRNA with a concomitant increase in the mRNA levels of pro-apoptotic Bcl-xS and Caspase 9a in several cancer cells. Our study thus identifies a novel mechanism of antitumor action for HHT (Fig. 7).

Phosphatase inhibitors calyculin A and okadaic acid were used to investigate the role of PP1 in HHT-induced alternative splicing. Calyculin A is an inhibitor of both PP1 and PP2A, while okadaic acid selectively inhibits PP2A. Our results showed that calyculin A, but not okadaic acid, significantly inhibits the effects of HHT on the alternative splicing of Bcl-x in MCF7, A549 and UACC903 cells. In MCF7 cells, HHT regulated the alternative splicing of Caspase 9 through a similar mechanism. To investigate the role of PP1 in HHT-mediated alternative splicing, we generated a stable MCF7 cell line overexpressing PP1. Overexpression of PP1 resulted in a decrease in the ratio of Bcl-xL/xS and an increase in the ratio of Caspase 9a/9b. Importantly, the effects of HHT on Bcl-x and Caspase 9 splicing were further enhanced in response to PP1 overexpression. These results indicated that HHT-induced alternative splicing of Bcl-x and Caspase 9 depends on the activation of PP1. Several studies have demonstrated other small molecules, such as ceramide and emetine, could regulate the alternative splicing of Bcl-x and Caspase 9 through a PP1-mediated splicing mechanism [15–17]. The effects of amiloride on the alternative splicing of both Bcl-x and HIPK3 might partially be mediated by PP1 through the dephosphorylation of SR proteins [18, 19]. Lamond and co-workers showed that dephosphorylation of SR proteins with PP1 induced alternative 5′ splice site selection in vitro [34]*.* SR proteins belong to a family of arginine–serine-rich domain containing proteins that are required for alternative splicing. The dephosphorylation of SR proteins with PP1 is critical to the splicing reaction [35, 36]. Future studies are needed to investigate the role of SR proteins in HHT-induced alternative splicing.

Previous studies have demonstrated that ceramide increases the pro-apoptotic Bcl-xS and Caspase 9a isoforms by regulating alternative splicing in A549 cells [15]. Consistent with this finding, emetine regulated alternative splicing of Bcl-x, increasing the pro-apoptotic Bcl-xS isoform and decreasing the anti-apoptotic Bcl-xL isoform [16]. However, emetine had an opposite effect on the alternative splicing of Caspase 9 in different tumor cell lines. In PC3 cells, emetine increased pro-apoptotic Caspase 9a with a concomitant decrease of anti-apoptotic Caspase 9b, while emetine increased anti-apoptotic Caspase 9b with a decrease of the pro-apoptotic Caspase 9a in C33A and MCF-7 cells [17]. In this study, HHT exhibited a cell type-specific effect on Caspase 9 splicing. HHT induced a significant increase in the ratio of Caspase 9a/9b in MCF7 and UACC903 cells, but had no effect on Caspase 9 splicing in A549 cells. These results suggest that HHT may mediate the alternative splicing of Bcl-x and Caspase 9 via different mechanisms. In accordance with this hypothesis, PP2A inhibitor okadaic acid partially relieved the effects of HHT on Caspase 9 splicing, but had no effect on Bcl-x splicing in MCF7 and UACC903 cells. It will be very interesting to address whether or not PP2A is involved in the HHT-induced alternative splicing of Caspase 9 in the future.

## Conclusions

Homoharringtonine regulates the alternative splicing of Bcl-x and Caspase 9, resulting in a decreased expression of anti-apoptotic Bcl-xL and Caspase 9b with a concomitant increase in the levels of pro-apoptoticBcl-xS and Caspase 9a in various cancer cells. Furthermore, the effect of HHT on alternative splicing is mediated by PP1. This study reveals a novel mechanism underlying the antitumor activities of HHT.

## Abbreviations

7-AAD: 7-Aminoactinomycin D
AML: Acute myeloid leukemia
cDNA: Deoxyribonucleic acid
CML: Chronic myeloid leukemia
DMEM: Dulbecco’s modified Eagle’s media
FBS: Fetal bovine serum
HHT: Homoharringtonine
MM: Multiple myeloma
PP1: Protein phosphatase 1
PP2A: Protein phosphatase 2A
RPMI1640: Roswell Park Memorial Institute 1640
Semi-quantitative RT-PCR: Semi-quantitative reverse transcriptase-polymerase chain reaction
